# Development of Miniprotein-Type Inhibitors of Biofilm Formation in *Candida albicans* and *Candida auris*

**DOI:** 10.4014/jmb.2411.11076

**Published:** 2025-02-25

**Authors:** Doyeon Kim, Ji-Seok Kim, Xue Bai, Jie Zhang, Minho Park, Ungyu Lee, Jinwook Lee, Yong-Sun Bahn, Yongbin Xu, Nam-Chul Ha

**Affiliations:** 1 Research Institute of Agriculture and Life Sciences, Department of Agricultural Biotechnology, CALS, Seoul National University, Seoul 08826, Republic of Korea; 2 Department of Biotechnology, College of Life Science and Biotechnology, Yonsei University, Seoul 03722, Republic of Korea; 3 Department of Bioengineering, College of Life Science, Dalian Minzu University, Dalian 116600, China; 4 Hanmi Pharmaceutical Co., Ltd., Seoul, Republic of Korea; 5 Center for Food and Bioconvergence, Department of Agricultural Biotechnology, Interdisciplinary Programs in Agricultural Genomics, CALS, Seoul National University, Seoul 08826, Republic of Korea

**Keywords:** Proteindesign, AI-driven protein engineering, *Candida auris*, antifungal

## Abstract

*Candida auris* is a pathogenic fungus associated with high-mortality infections and forms resilient biofilms on various surfaces. In this study, we introduced a novel antifungal strategy against *C. auris* by integrating an AI-powered protein design tool, ProteinMPNN, with classical molecular dynamics (MD) simulations to design artificial proteins from a miniprotein library. This combined approach accelerated and enhanced the design process, enabling the rapid development of effective miniprotein inhibitors specifically targeting *C. auris* biofilm formation. The miniproteins developed in this study exhibited potent inhibitory effects on *C. auris* biofilms, representing a significant advancement in antifungal therapy. Notably, the combined application of these miniproteins enhanced suppression of biofilm formation. These findings highlight not only the strong therapeutic potential of these designed miniproteins but also the power of combining AI-driven protein design with MD simulations to advance biomedical research.

## Introduction

The integration of artificial intelligence (AI) into protein engineering has led to a transformative era in protein design with far-reaching implications for research and pharmacology [1, 2]. AI-driven tools facilitate the development of protein binders for target proteins, akin to monoclonal antibodies, which are vital for fundamental research and the generation of novel therapeutics. Among these approaches, *de novo* protein design has gained prominence, offering the ability to identify unique protein binders specific to receptor proteins.

Advances in deep learning have markedly improved the ability to predict protein structures. Programs such as AlphaFold [3] and RosseTTAfold [4], powered by deep learning algorithms, can reliably predict the three-dimensional structures of proteins based on their amino acid sequences [3, 4]. Additionally, ProteinMPNN [5], another deep learning-driven tool, helps predict amino acid sequences from predetermined protein structure frameworks, with its effectiveness validated through both computational modeling and empirical studies [6]. ProteinMPNN demonstrates great potential in engineering proteins that specifically bind to target proteins, as it predicts sequences within multi-chain protein complexes while maintaining the sequence integrity of the target receptors.

Despite the groundbreaking advances offered by AI-based tools, traditional computational methods remain indispensable and serve as complementary approaches to AI innovations. Molecular dynamics (MD) simulations [7] are particularly notable in this regard. MD simulations offer a dynamic perspective by simulating protein behavior under various thermal and pressure conditions, providing insights into protein stability and flexibility [7]. These simulations complement AI-based predictive binding models by revealing conformational shifts that can refine and validate binding prediction.

*Candida albicans* and *Candida auris* are both pathogenic yeasts capable of causing infections in humans, particularly in immunocompromised individuals [8, 9]. *C. auris* exhibits high antifungal resistance in some clades [10, 11]. The antifungal resistance of *C. albicans* are increasing in certain regions although the majority of *C. albicans* clinical isolates worldwide remain sensitive to antifungal agents. For example, fluconazole resistance in *C. albicans* has been reported in approximately 3–8% of global isolates [12].

Both species are known for their ability to form resilient biofilms, which enhance their survival and complicate treatment efforts [13]. *C. auris* biofilms, in particular, exhibit higher resilience and adaptability in clinical settings, contributing to significant challenges in infection control. *C. auris* can cause a spectrum of infections ranging from superficial infections, such as wound or ear infections, to severe systemic infections like bloodstream infections (candidemia). Patients infected with *C. auris*, especially those with weakened immune systems, face high mortality rates [14, 15].

In *C. albicans*, biofilm formation is facilitated by virulence factors such as the agglutinin-like sequence (ALS) protein family, which plays a central role in cell adhesion and biofilm development. The ALS gene in *C. albicans* includes *ALS1-ALS7* and *ALS9*. The Als proteins are divided into four distinct domains: the N-terminal (NT) domain, T domain, central tandem repeat (TR) domain, and C-terminal (CT) domain. The TR domain, characterized by a repeated arrangement of serine and threonine residues, is central to the protein’s structure and serves as a key criterion for differentiating the subfamilies within the ALS family proteins [16]. The NT domain is directly involved in the adhesion of Als proteins to various substrates and contains two immunoglobulin-like (Ig-like) domains with a peptide-binding cavity (PBC) between them [17, 18]. Additionally, a highly conserved amyloid-forming region (AFR) lies between NT and T domain, and is predicted to facilitate binding between Als proteins [19]. The T domain serves as a connector between the NT and TR domains, though its precise function and structure remain poorly understood. The CT domain contains a glycosylphosphatidylinositol (GPI) anchor addition sequence, which is crucial for anchoring Als proteins to the fungal cell wall [20].

Miniprotein scaffold libraries, such as those developed by Long *et al*. [21], represent a diverse collection of protein frameworks characterized by their small size (50–60 amino acids), remarkable stability, and potent drug-like properties. These miniproteins are particularly well-suited for biomedical applications, serving as inhibitors of protein-protein interactions or as agonistic and antagonistic drugs targeting specific receptor proteins. Their unique attributes make them promising candidates for therapeutic development, especially in challenging contexts where traditional small molecules or biologics may fall short.

Through extensive research, we identified significant sequence and structural homology between the ALS family proteins of *C. albicans* and *C. auris*. This discovery highlights Als proteins as central targets for antifungal therapy. In this study, we introduced a novel application of miniprotein design to develop specific inhibitors targeting *C. auris* biofilms. By leveraging the synergistic capabilities of ProteinMPNN and MD simulations, we successfully engineered miniproteins to disrupt the biofilm formation process in *C. auris*, representing a significant step forward in developing innovative antifungal therapies.

## Material and Methods

### Screening Binding Proteins from the Miniprotein Library Using PatchDock Module

The miniprotein library was downloaded from https://files.ipd.uw.edu/pub/robust_de_novo_design_minibinders_2021/supplemental_files/scaffolds.tar.gz [21]. PatchDock [22] was used to identify potential miniproteins that could bind to the target protein using a bash script (patchDockMulti). To parallelize this process, the 'parallel' command was implemented in a bash script (patchdock20). A separate bash script (crmsd) was used to select miniproteins that bound to a specific site of interest on the target protein.

### Obtaining the Amino Acid Sequence Using ProteinMPNN Module

Using the coordinates of the miniprotein and target protein complex, the amino acid sequence of the miniprotein in complex with the target protein was determined using ProteinMPNN [5] in a script (mpnn). The generated sequences were evaluated for accurate 3D structure formation using ESMFold [6]. Sequences with a pLDDT value below 0.7 were discarded. The structures generated by ESMFold were superimposed onto the miniprotein structure input to ProteinMPNN using pTM-align [24], and RMSD values were calculated. Structures with RMSD values greater than 1.0 Å were rejected. Finally, the complex structure of the ProteinMPNN-generated miniprotein and target protein was obtained. Thirty complex structures were selected for the next step by visual inspection using PyMol [23].

### Binding Analysis of Energy Minimized Complexes Using GROMACS Module

The complex structures obtained through the ProteinMPNN module were energy-minimized using the GROMACS [25], and their binding scores were evaluated using, PRODIGY [26]. Subsequently, an MD simulation for 10-ns was conducted using GROMAC, and the resulting MD simulated complex structure was assessed using PRODIGY. Finally, the binding scores of the initial energy-minimized complex and MD simulated complex were compared. The absolute scores and improvements in scores owing to the MD simulation were analyzed to select the final three candidates.

### BLAST Analysis of *C. albicans* ALS3, *C. albicans* ALS9 in *C. auris*

To identify *C. albicans* ALS homologs in *C. auris*, the Basic Local Alignment Search Tool for Proteins (BLASTp) was used. The N-terminal domain sequence of *C. albicans* Als3 protein was derived from the crystal structure (PDB code: 4LE8), covering residues 18-316 [27]. The N-terminal domain sequence of *C. albicans* Als9 protein was obtained from its crystal structure (PDB code: 2Y7L), covering residues 18–329 [18]. These sequences were used as queries to search for similar sequences in *C. auris* (taxid: 498019), identifying homologous sequences for *C. albicans* Als proteins in *C. auris*. To compare with the N-terminal domains of CaAls3 and CaAls9, the sequence of CJJ09 (residues 1–329) was aligned and its sequence similarity was calculated.

### Plasmid Construct and Protein Expression of the Miniproteins

The sequences of the designed miniproteins Als3_1224, Als3_3743, and Als9_1390 were codon optimized for expression in *E. coli* and synthesized by Gene Synthesis Services (Sangon Biotech, China) (Table 1). All miniprotein sequences were cloned into the pET28a vector (Merck, USA) using the NcoI and XhoI restriction sites. The recombinant miniprotein constructs were transformed into *E. coli* BL21(DE3) cells for protein overexpression and purification. Transformed cells were cultured in 1.5 L of LB medium containing 50 μg/ml kanamycin at 37°C until the optical density at 600 nm (OD_600_) reached 0.6. Protein overexpression was induced by adding 0.5 mM isopropyl-β-D-thiogalactoside (IPTG) to the medium, followed by incubation at 30°C for 6 h. The cells were then harvested by centrifugation at 5,500 ×*g* and stored at -80°C in a deep freezer until further purification.

### Purification of the Miniproteins

Harvested *E. coli* cells expressing each miniprotein were resuspended in 50 ml lysis buffer (20 mM Tris-HCl pH 8.0, 150 mM NaCl) and lysed using a French press at 23 kpsi. The cell lysate was then centrifuged at 13,000 rpm for 30 min at 4°C to remove cell debris. The clarified lysate was incubated with Ni-NTA agarose resin (1 ml; Qiagen, Germany) by rolling at 4°C for 1 h. Unbound materials were removed from the resin by washing with 250 ml of wash buffer (20 mM Tris-HCl pH 8.0, 150 mM NaCl, 20 mM imidazole pH 8.0). Bound proteins were subsequently eluted with 20 ml of elution buffer (20 mM Tris-HCl pH 8.0, 150 mM NaCl, 250 mM imidazole pH 8.0). Subsequently, anion exchange chromatography was performed using a 5 ml HiTrap Q HP column (Cytiva) with a gradient from 0 mM to 1 M NaCl for elution. Als3_1224 was eluted at 300–450 mM NaCl, and Als9_1390 eluted at 200–350 mM NaCl, while Als3_3743 did not bind to the column (Fig. S1). Each eluted fraction or unbound solution was concentrated to a final volume of 5 mL using a Millipore centrifugal concentrator (3 kDa molecular weight cutoff; Millipore, USA). The purified protein solution was subsequently concentrated with a Millipore centrifugal concentrator (3 kDa molecular weight cutoff; Millipore) and stored at -80°C until further use.

### Crystal Violet Biofilm Quantification Assay

Cells of *C. auris* (B8441), *C. albicans* (SC5314), and *C. neoformans* (H99) wild-type strains were cultured overnight at 30°C in 2 ml YPD liquid medium, washed twice with H_2_O, and resuspended in MOPS-buffered RPMI-1640 media (pH 7.4 with 0.165 M MOPS and 2% glucose). For the crystal violet assay, the cell suspension was prepared with a concentration equivalent to an OD_600_ of 0.5. Subsequently, 200 μl of the cell suspension was loaded into each well of a 96-well plate, each inhibitor was treated at a concentration of 10 μM, and the cultures were incubated at 37°C and 220 rpm for 1 day. The next day, the cell suspension was removed, and the samples were completely dried in a dry oven at 65°C. To each well, 150 μl of a 0.3% crystal violet solution was added, and the samples were stained at room temperature for 10 min. Subsequently, the crystal violet solution was completely removed, and the wells were washed three times with ddH_2_O. The samples were dried in an oven at 65°C. Next, 200 μl of 33% acetic acid was added to each well, and the crystal violet was dissolved completely for 1 min. The solution was transferred to a clean 96-well plate, and the absorbance was measured at OD_595_.

For the experiments involving *C. auris* biofilm treated with miniproteins at concentrations ranging from 1 nM to 5 μM, as well as in double and triple combinations, *C. auris* cells were cultured and harvested using the same method described earlier. And 200 μl of the cell suspension was loaded into each well of a 96-well plate. Subsequently, each inhibitor was tested at concentrations of 1 nM, 10 nM, 100 nM, 1 μM, 2 μM, and 5 μM. For combination treatments, each miniprotein was applied at a concentration of 1 μM. The quantification of biofilm formation using crystal violet staining was conducted as previously described.

## Results

### A Proposed Workflow for Identifying Miniproteins for Receptor Proteins

To develop protein binders, we employed miniproteins [21] as the foundational scaffolds. Miniproteins are designed to adopt specific, stable folds; however, they lack inherent binding specificity. Therefore, a specialized workflow is essential to engineer these miniproteins into receptor-specific binders with high affinity and selectivity.

**Fig. 1:**
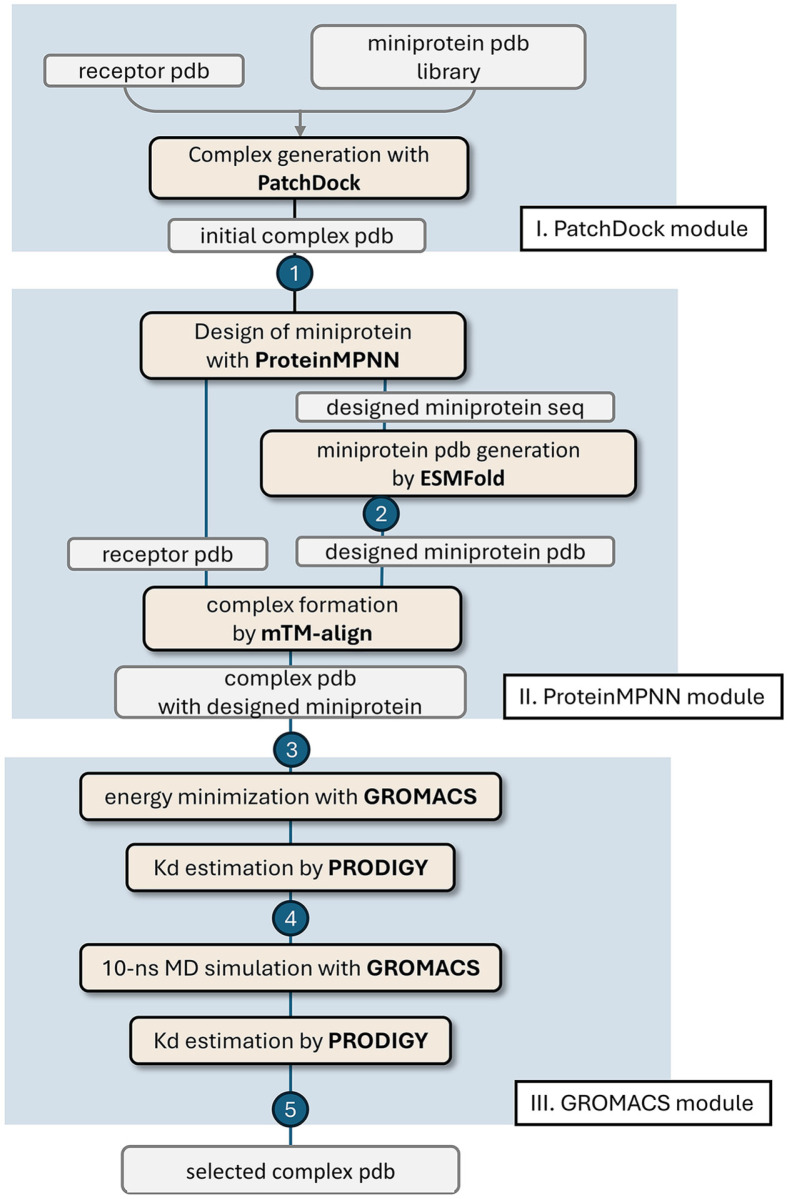
Workflow for the Design and Selection of Miniprotein Binders. The workflow is divided into three modules: PatchDock, ProteinMPNN, and GROMACS module. The five Checkpoints are indicated as numbers in the circles. All scripts used in this study are available at https://github.com/snufoodbiochem/miniprotein_design/tree/master/miniscripts_ver0.1 (**A**) PatchDock module: The target receptor protein and a miniprotein library consisting of approximately 26,000 PDB files were input into PatchDock [22], which generated initial complex PDB files with five complexes produced per miniprotein using script patchdock20. Complexes in which the miniprotein bound to the receptor protein with a high binding score at the site of interest were selected at Checkpoint 1. (**B**) ProteinMPNN module: The complex PDB files were used as inputs for ProteinMPNN [5]. The receptor PDB is designated as the fixed chain, while the miniprotein is treated as the designed chain, generating the "designed miniprotein sequences" optimized for receptor binding. To verify whether the ProteinMPNNgenerated sequences could form the intended miniprotein structures, ESMFold [6] was utilized (scripts mpnn and ef_run). Sequences with a pLDDT value of less than 0.7 in ESMFold are rejected at Checkpoint 2. The designed miniprotein PDB files were combined with the receptor PDB file using the structural alignment program mTM-align [24], resulting in a complex PDB files containing the designed miniproteins using the script ef_align. Complex PDB files were rejected if the binding mode or position changed significantly (for example, RMSD values > 1.0 Å). (**C**) GROMACS module: The complex PDB files were input into the MD simulation program GROMACS (gmx) [25]. During MD simulation, the complex PDB files underwent energy minimization to determine the relaxed conformation at the binding interface. The PRODIGY program [26] was used to estimate the binding affinity energy (Kd) values. Complex PDB files with Kd values higher than 10^-6^ are rejected at Checkpoint 4. 10 ns MD simulations were performed on the energy-minimized PDB file using GROMACS (gmx). PRODIGY was used to estimate the binding energies or Kd values. Complex PDB files were rejected at Checkpoint 5 if the binding affinity decreased after the 10 ns MD simulation. Finally, the designed miniproteins in the complex were visually inspected using PyMOL [23], and the PDB files of one to three candidates were selected in the final step.

To address this, we propose a workflow consisting of three consecutive modules: PatchDock, ProteinMPNN, and GROMACS. The first step, the PatchDock module, generates initial complex PDB files of receptor proteins and miniproteins, which have not yet been optimized for binding. PatchDock, a tool developed 20 years ago, primarily searches for docking solutions based on shape complementarity between ligands and receptor proteins, excluding electrostatic interactions [22]. This approach is particularly suitable at this stage because the miniproteins are still unrefined for binding to the target receptor. For this module, we randomly selected approximately 5,000 miniproteins from the scaffold library to use as ligand proteins in PatchDock. For each miniprotein, the top five docking solutions were ranked based on the distance between the center of mass of the miniprotein and the target site on the receptor protein. To refine the results, we prescreened the complex structures based on both distance and PatchDock scores, reducing the dataset to around 200 complexes. At this stage, called Checkpoint 1, we examined the structures using PyMoL [23] to ensure their viability for further optimization. This structural review resulted in the selection of approximately 100 complexes, which were progressed to the next module (Fig. 1).

The second module, ProteinMPNN module, involved the sequential execution of ProteinMPNN [5] and ESMFold [6], and mTM-align [24] to optimize the sequences of the miniproteins binding to the receptor proteins. The sequences of the miniproteins in the complex generated by Checkpoint 1 were refined using the AI-driven program ProteinMPNN [5]. This step was automated using a bash script, which specified the chain ID of the ligand miniprotein for sequence design while keeping the receptor protein unchanged. After generating optimized sequences, we validated whether the predicted sequences of the miniprotein retained the original backbone structure using ESMFold [6], a protein structure prediction tool based on a large language model. Proteins with pLDDT (predicted Local Distance Difference Test) values below 0.7 were rejected at Checkpoint 2 (Fig. 1), as these values indicated insufficient structural confidence. Because ESMFold resets the origin and orientation of the PDB file during prediction, we realigned the miniprotein PDB files with the original complex PDB files from the initial PatchDock results. This realignment was performed using mTM-align [24]. Complexes showing significant differences between the original and the predicted miniproteins were rejected at Checkpoint 3 (Fig. 1).

In the final module, we incorporated MD simulations using the GROMACS program [25] to assess the strength of protein-protein interactions. MD simulations, combined with AI-driven protein-prediction tools, play pivotal roles in protein engineering by providing insights into protein functions and behaviors. The energy of the complex structure, comprising the receptor and ligand proteins generated by ESMFold, was minimized using the GROMACS program. Subsequently, we conducted a 10 ns MD simulation to study the dynamics of the complex. The binding strengths of the energy-minimized structure and the structure obtained after the 10 ns MD simulation were then compared. We utilized the predicted dissociation constant (Kd) values, calculated using PRODIGY [26], and the changes in Kd values after the MD simulation to assess the binding abilities of the designed miniprotein binders (Fig. 1). This analysis provided critical metrics for evaluating the efficacy of the miniproteins in formatting stable interactions with their target receptors.

### Sequence Similarity of *C. auris* *CJJ09* to NT Domain of *C. albicans* Als3 and Als9 Proteins

**Fig. 2:**
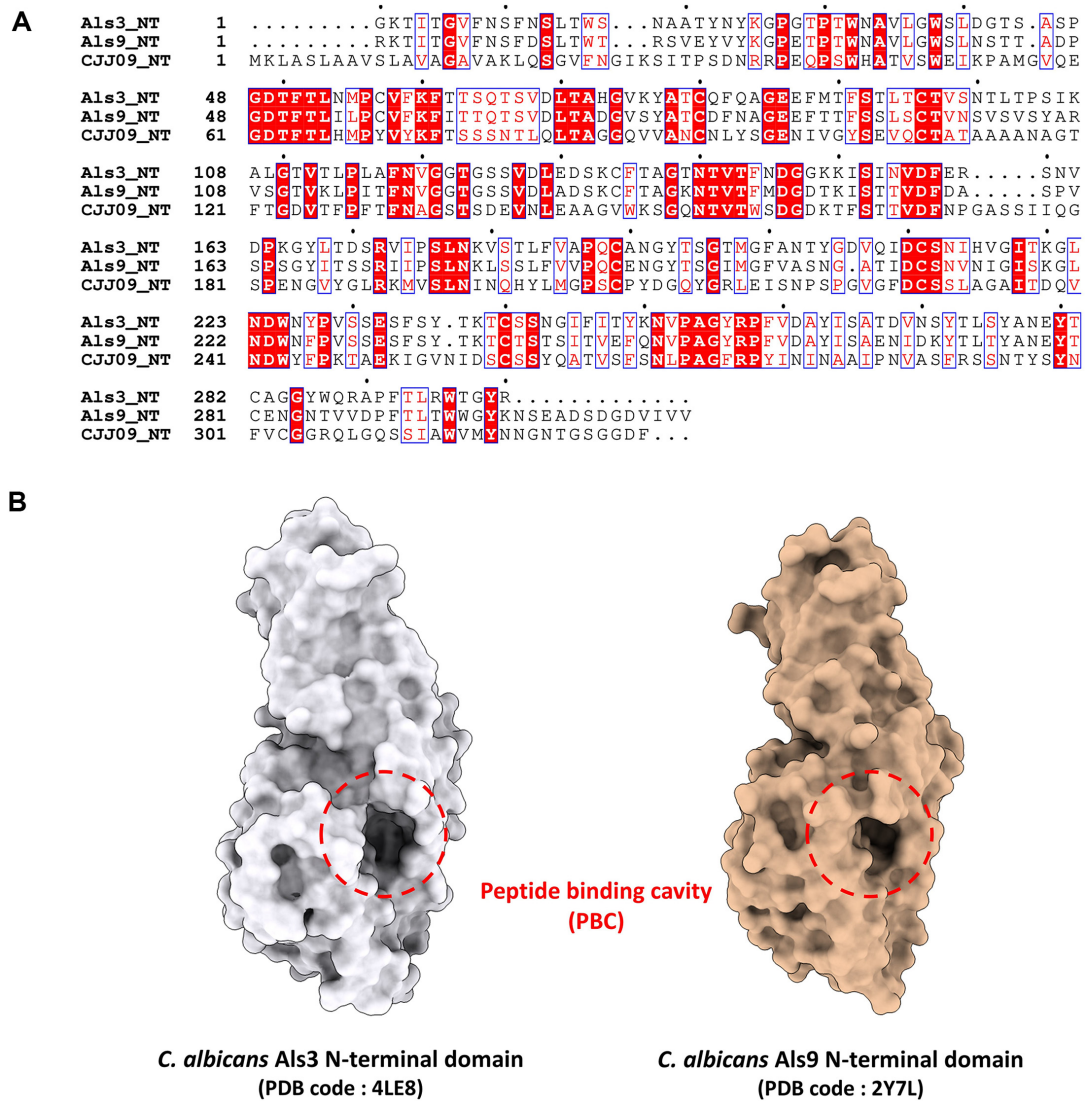
Amino acid sequences and the peptide binding cavities (PBC) of Als3 protein and Als9 protein from *C. albicans*. (**A**) Sequence alignment of the N-terminal domains of Als3 and Als9 from *C. albicans* and the N-terminal domain of the *ALS3/ALS9* homologue (CJJ09) from *C. auris*. Sequence alignment was performed using ClustalX [38] and ESPript server [39]. (**B**) Crystal structures of the N-terminal domains of Als3 (PDB code: 4LE8 [27]) and Als9 (PDB code: 2Y7L [18]). The PBC is indicated by the circles in the broken lines.

*C. auris* possesses *ALS* homologs that may share similar structural features with those in *C. albicans*, although further studies are required to fully elucidate these functions. Our analysis identified that the NT domain of CJJ09_005316 (called CJJ09 in this study) from *C. auris* exhibits the highest sequence similarity to the corresponding domain of *C. albicans* Als3 protein (Fig. 2A) [27]. Similarly, the NT domains of CJJ09 from *C. auris* showed the highest sequence similarity to the NT domain of *C. albicans* Als9 protein (Fig. 2B) [18]. The alignment of the NT sequences revealed that CJJ09 shares 74.0% similarity with the N-terminal Als3 protein, and 73.9%similarity with the N-terminal Als9 protein. These findings establish CJJ09 as ALS homologs in *C. auris*, potentially performing roles analogous to *ALS3* and *ALS9* in *C. albicans*.

### Design of Miniproteins for Binding *C. albicans* Als3 Protein

To inhibit biofilm formation by *C. albicans* and *C. auris*, we focused on the Als3 and Als9 proteins from *C. albicans* to develop specific protein binders. Als3 protein plays crucial roles in *C. albicans*' adhesion to host cells, biofilm formation, and iron acquisition from the host, all of which are vital for its pathogenicity. The NT domain of Als proteins, which is highly conserved across *Candida* species, is directly involved in adhesion to host cells [20, 28]. Given its functional importance, we selected Als proteins as targets for miniprotein design, focusing on the structure and sequence of their NT domain.

The crystal structure of the wild-type Als3 protein from *C. albicans* (CaAls3, PDB code: 4LE8) has been used, which encompasses the sequence of the NT domain (residues 1-299) [27]. A key feature of the PBC in the protein is an invariant lysine residue at its terminus, which stabilizes interactions by forming a salt bridge with the C-terminal carboxyl group of peptide ligands. Previous studies have underscored the importance of PBC in facilitating the adhesion of *C. albicans* to host cells and other abiotic surfaces (Fig. 2B) [27, 29].

To design miniproteins, we used PatchDock [22] to dock CaAls3 with 2,000 randomly selected members from a miniprotein library. Based on the PatchDock scores, we selected the top 200 miniprotein sequences that are bound to the PBC. We then optimized these sequences using ProteinMPNN [5], fixing CaAls3 to the binary complexes. The resulting sequences were confirmed by ESMFold [6], and miniproteins with root-mean-square deviation (RMSD) values exceeding 1 Å (as determined by mTM-align [24]) were excluded.

**Fig. 3:**
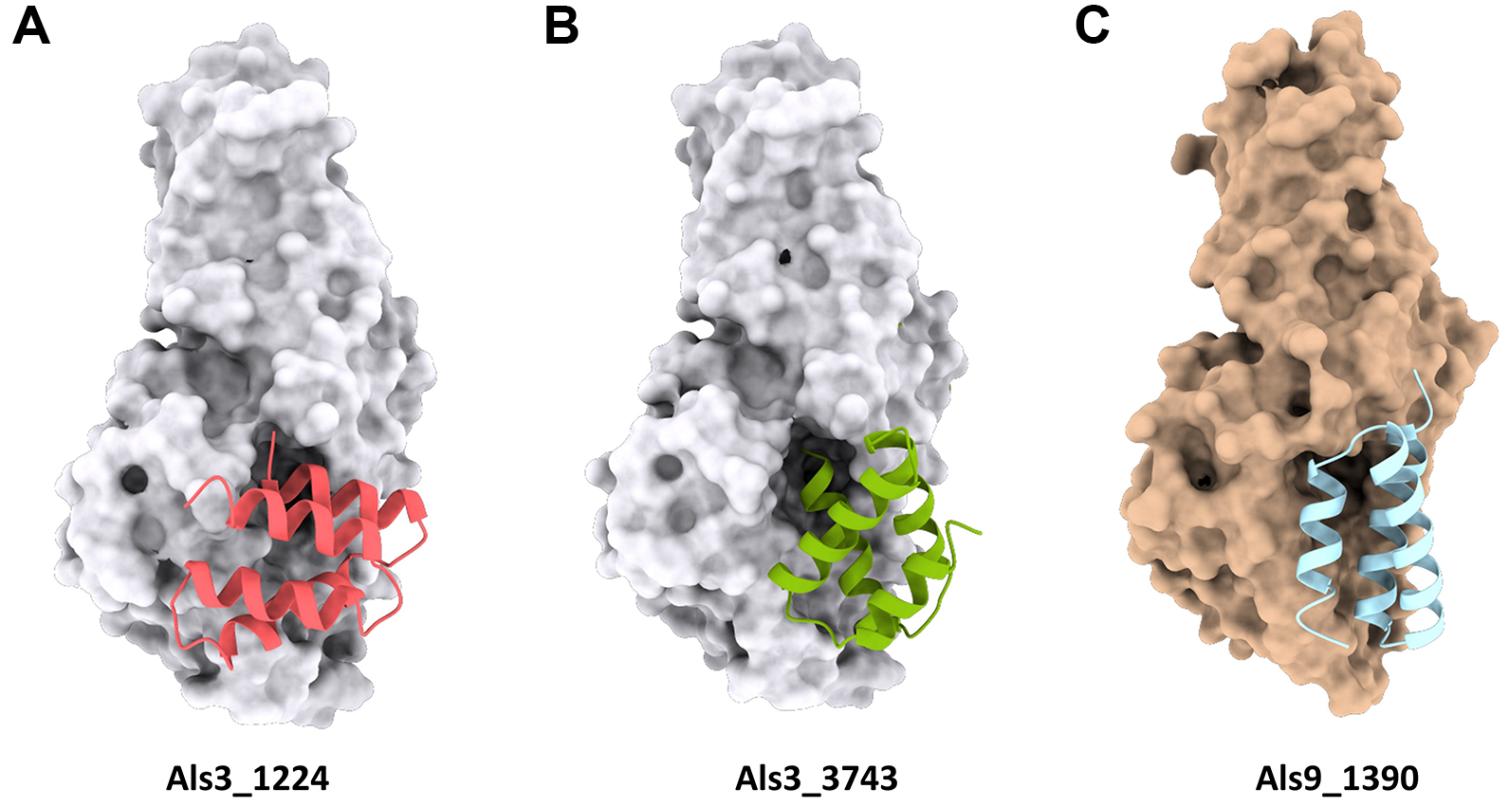
The complex models of the Als3 (pink, green) and Als9 (bright blue) N-terminal domain and their miniprotein binders. The complex models were built by structural superposition based on the GROMACS output. (**A**) The miniprotein binder Als3_1224 (pink) binds to the PBC of Als3 from *C. albicans*. (**B**) The miniprotein binder Als3_3743 (green) binds to the PBC of Als3 from *C. albicans*. (**C**) The mini-protein binder Als9_1390 (bright blue) binds to the PBC of Als9 from *C. albicans*.

Next, MD simulations were performed using GROMACS program [25] to refine the CaAls3-miniprotein complexes. The complex structures were energy-minimized, equilibrated, and subjected to a 10 ns MD simulation. We then compared the estimated Kd values between the energy-minimized and MD-simulated structures, using PRODIGY [26]. Complexes with Kd values exceeding 10^-9^ M or those whose Kd values increased after MD simulation were excluded. After thoroughly reviewing the complex structures, we identified two promising candidates: Als3_1224 and Als3_3743 (Fig. 3A and 3B). These miniproteins are expected to function as strong potential inhibitors of CaAls3, targeting their role in biofilm formation and pathogenicity.

### Design of Miniprotein for Binding *C. albicans* Als9 Protein

The Als9 protein contributes to adhesion in *C. albicans*, although its role is a less prominent role than Als3 protein [28, 30]. Given the known crystal structure of *C. albicans* Als9 protein, we sought to develop miniproteins targeting this structure. As *C. auris* has a homologous counterpart to *C. albicans* Als9 protein, these miniproteins may also effectively disrupt biofilm formation in *C. auris*.

Using the same approach as in designing CaAls3 binding miniproteins, we designed miniproteins targeting the NT domain of the Als9 protein from *C. albicans* (CaAls9, PDB code: 2Y7L) [18]. The CaAls9 NT domain structure used in this study included a bound fibrinogen-γ peptide. To create miniproteins that inhibit the binding of this peptide binding PBC, we omitted the bound fibrinogen-γ peptide from the structure and designed binders targeting the PBC. Through PatchDock module, we selected top miniproteins from our library based on their binding affinities. Miniproteins that effectively blocked access to the PBC were prioritized for further analysis. After identifying the optimal binding positions, we generated amino acid sequences for the miniproteins using the ProteinMPNN module. Structural refinement and validation were carried out using ESMFold [6], mTM-align [24], and GROMACS program [25] from the GROMACS module, as previously described. Miniproteins with a PRODIGY [26] score greater than 10^-9^ M or those whose Kd values increased after MD simulation in the GROMACS module were excluded. Following a thorough examination of the complexes, we selected miniprotein called Als9_1390 as the final candidate due to its exceptional performance. This miniprotein exhibited a Kd value of 0.18 nM after a 10-ns MD simulation, highlighting its strong binding affinity to CaAls9 (Fig. 3C).

### Biofilm Formation Inhibition in Pathogenic Fungi by Designed Miniproteins

The effects of Als proteins inhibitors on biofilm formation were assessed using a crystal violet biofilm quantification assay [31] in three major pathogenic fungi: *C. albicans* (SC5314), *C. auris* (B8441), and *C. neoformans* (H99). Biofilm formation was quantified after treatment with the Als protein inhibitors Als3_1224, and Als3_3743, and Als9_1390, each at a concentration of 10 μM.

**Fig. 4:**
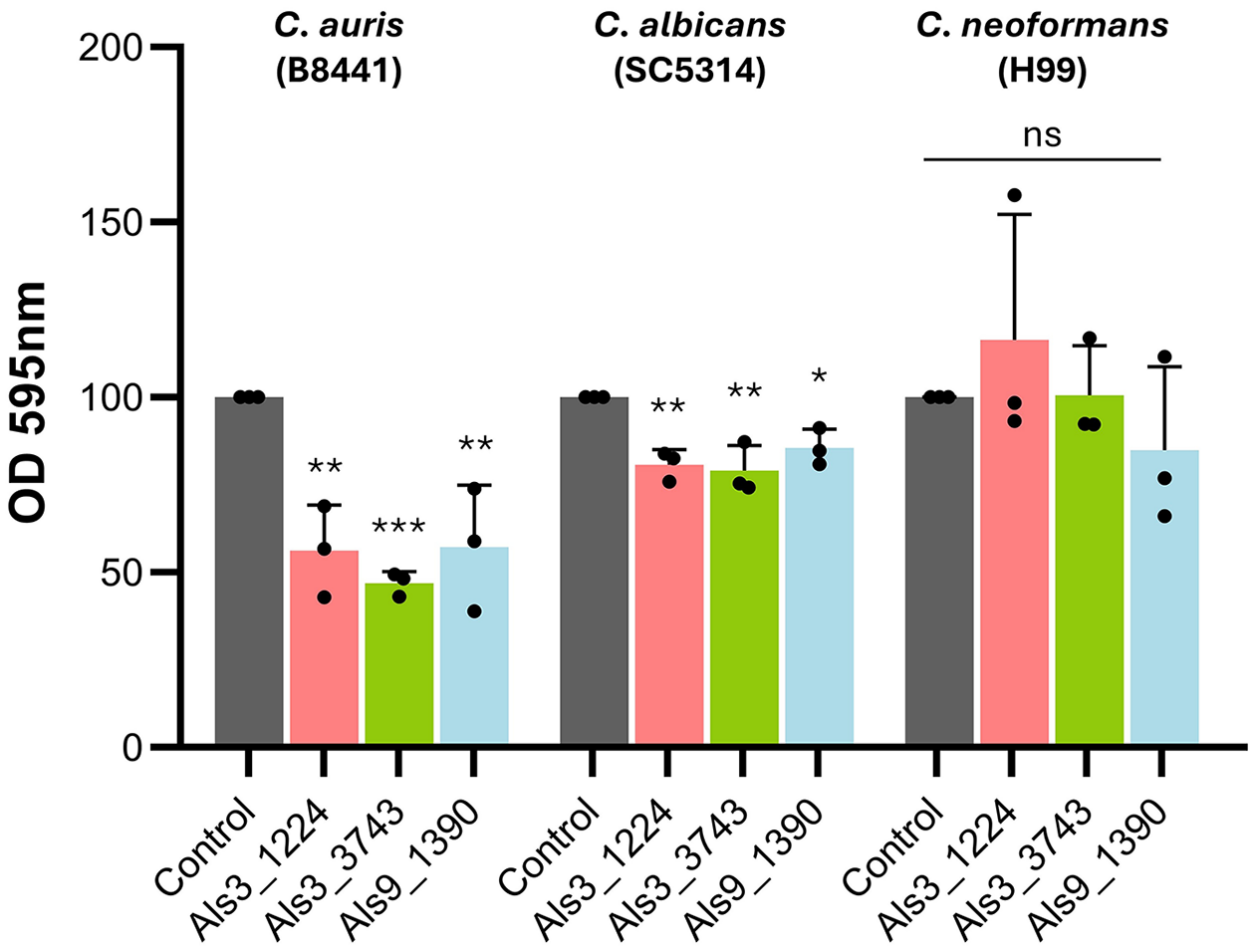
Inhibition of biofilm formation in major pathogenic fungi by Als inhibitors. Biofilms of *C. auris*, *C. albicans*, and *C. neoformans* were treated with Als3_1224, Als3_3743, and Als9_1390 (10 μM) for 24 h, followed by crystal violet staining. The absorbance of the destaining solution was measured at 595 nm under all treatment conditions. Statistical analysis was conducted using a one-way ANOVA with Bonferroni’s multiple comparison test against the control group (* *p* < 0.05; ** *p* < 0.01; *** *p* < 0.001; **** *p* < 0.0001).

The Als protein inhibitors significantly reduced biofilm formation in both *C. auris* and *C. albicans* (Fig. 4). However, these inhibitors had no significant effect on *C. neoformans*, which lack *ALS* genes. Interestingly, the miniproteins designed for Als proteins in *C. albicans* demonstrated greater efficacy in inhibiting *C. auris* biofilms. We hypothesized that the target of these miniproteins is likely CJJ09, given their sequential similarity of CaAls3 and CaAls9.

**Fig. 5:**
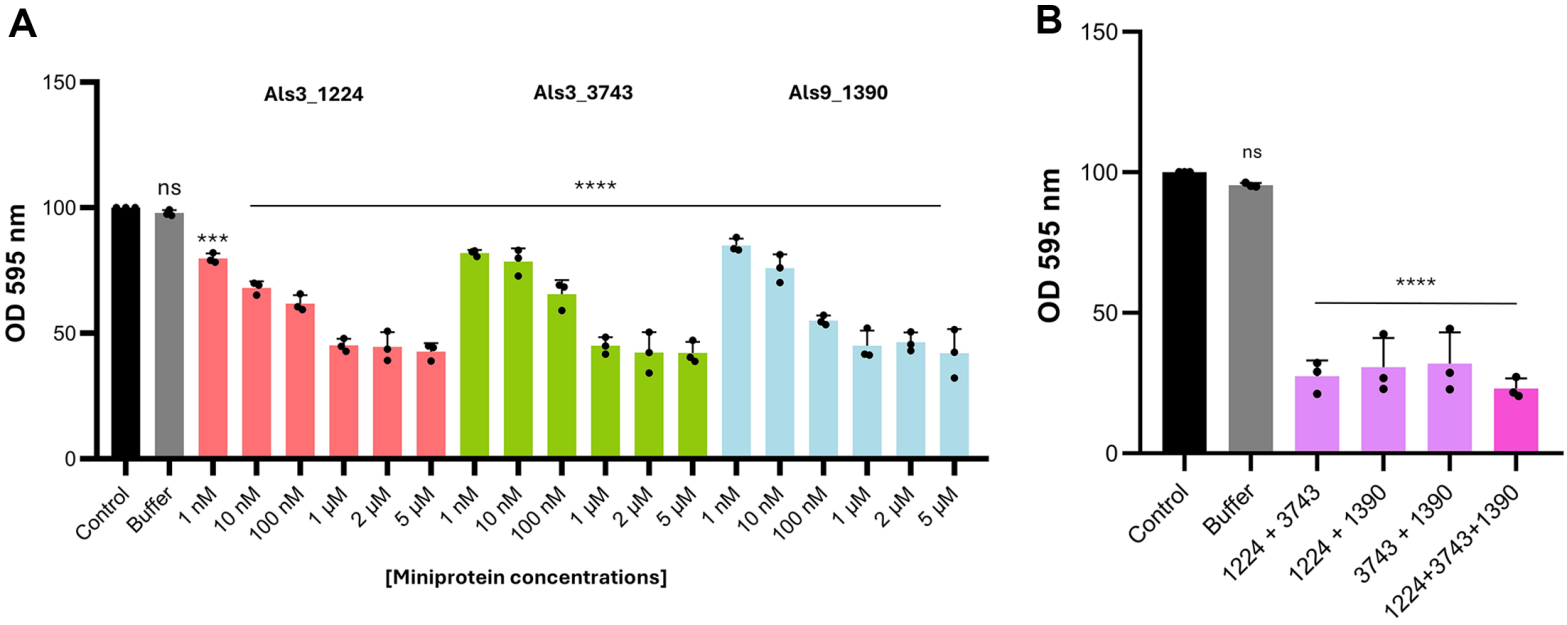
Dose-dependent and additive effects of miniproteins on *C. auris* biofilm formation. (**A**) The dosedependent effects of miniproteins on *C. auris* biofilm formation. Biofilm formation was measured using crystal violet staining at various concentrations. (**B**) The effects of combinatorial treatments of miniproteins (1 μM each) on *C. auris*. Biofilm formation levels were assessed using crystal violet staining. Statistical analysis was conducted using one-way ANOVA with Bonferroni’s multiple comparison test against the control (*, *p* < 0.05; **, *p* < 0.01; ***, *p* < 0.001; ****, *p* < 0.0001).

To assess the inhibitory potency, the effects of the miniproteins on *C. auris* biofilm formation were tested across a range of concentrations. Increasing the concentration of the miniproteins led to progressively greater reductions in biofilm formation (Fig. 5A). Statistical analysis of miniprotein treatments ranging from 1 nM to 5 μM revealed that Als3_1224, Als3_3743, and Als9_1390 achieved 50% biofilm formation at a concentration of 1 μM. This concentration-dependent inhibition underscores the therapeutic potential of miniproteins in modulating *C. auris* biofilm development (Fig. 5A).

Given these promising results, we further examined the combined effects of the miniproteins on *C. auris* biofilm formation. Co-treatment with Als3_1224, Als3_3743, and Als9_1390 enhanced biofilm inhibition compared to individual treatments (Fig. 5B). While a single miniprotein treatment at 1 μM reduced biofilm formation by approximately 50%, the combination of two miniproteins at 1 μM each achieved a 70% reduction in biofilm formation. Moreover, the treatment combining all three miniproteins at 1 μM each resulted in an 80% reduction in biofilm formation. These findings suggest an additive effect of the miniproteins, highlighting the overlapping functions of Als proteins in *C. auris* biofilm formation. Our results indicate that blocking a single Als protein is insufficient to fully prevent biofilm formation in *C. auris*. However, simultaneously targeting multiple Als proteins significantly amplifies their inhibitory effect, making this a promising strategy for managing *C. auris* biofilm development.

## Discussion

Numerous methods have been developed to design proteins that specifically bind to target proteins [32-36]. Our study introduces a novel approach for selecting miniprotein binders from a miniprotein library and optimizing their protein sequences using programs like PatchDock [22], ProteinMPNN [5], ESMFold [6], and MD simulations. This integrated workflow enabled the development of miniprotein inhibitors targeting *C. auris* biofilm formation, demonstrating potent inhibitory effects. This combined approach significantly enhanced biofilm suppression and underscored the therapeutic potential of the designed miniproteins and the effectiveness of integrating AI-driven programs with MD simulations in biomedicine.

Interestingly, the designed miniproteins exhibited greater efficacy against *C. auris* than *C. albicans*. This difference may be attributed to variations in the polymorphic forms of these two species. *C. albicans* can exist in yeast, pseudohyphal, and true hyphal forms, which enhance their adaptability and pathogenic potential. This polymorphic nature allows *C. albicans* to survive and thrive under diverse environmental and host conditions including fluctuations in temperature, pH, oxygen, and carbon dioxide levels. Conversely, while *C. auris* can also transition between yeast and hyphal forms, its polymorphism is less pronounced, especially with respect to true hyphal formation [37]. This reduced morphological complexity may explain why the miniproteins were more effective against *C. auris*, although further studies are needed to confirm this observation.

Despite these promising results, current miniprotein design techniques face notable limitations. One major challenge lies in designing miniproteins with dissociation constants (*K*d) in the nanomolar range, which is critical for achieving high-affinity binding to target proteins. The inability to routinely design miniproteins with such strong binding affinities highlights a significant technological gap in the field. Advancing miniprotein design to overcome this limitation will require the development of new algorithms and methodologies, potentially incorporating enhanced molecular dynamics simulations, advanced AI models, or hybrid approaches that combine computational and experimental techniques.

Our findings align with the growing recognition of *C. auris* as a major fungal pathogen, primarily because of its high antifungal resistance and ability to spread in healthcare settings. These results provide valuable insights into the role of the Als protein family in *C. auris* and highlight the potential of using combinations of miniproteins as a more effective strategy to inhibit biofilm formation. This approach could pave the way for the development of novel therapeutic strategies targeting *C. auris* infections, which are notoriously difficult to treat with conventional antifungal therapies owing to the protective nature of biofilms.

In summary, the integration of AI-driven protein design with MD simulations represents a promising avenue for developing innovative antifungal agents. By targeting biofilm formation through precise miniprotein inhibitors, this approach offers a new direction for combating infections caused by *C. auris* and other biofilm-forming pathogens.

## Supplemental Materials

Supplementary data for this paper are available on-line only at http://jmb.or.kr.



## Figures and Tables

**Table 1:** Amino acid sequences for miniproteins.

Miniprotein	Sequence
Als3_1224	GGYYKLVGKAVELGLPVTELMALISQASAQAGGDATATLAILAELLEAAGYPELAALVREALASS
Als3_3743	GLMADLQNLLLMYQRTGDPEYLKKVAQLALKAAGSEAAAEKMIAELVATLGLPAEVEKALKALLK
Als9_1390	LQAGLQVTQLCIEALQLARTDGAAAKAKLAQAKAVATAANNPALVAKVDATGALL
