# Supplementary figures and images for: Development of Miniprotein-Type Inhibitors of Biofilm Formation in Candida albicans and Candida auris
Source: J Microbiol Biotechnol. 2025 Feb 25;35:e2411076. doi: 10.4014/jmb.2411.11076 (PMC11896806; doi:10.4014/jmb.2411.11076)

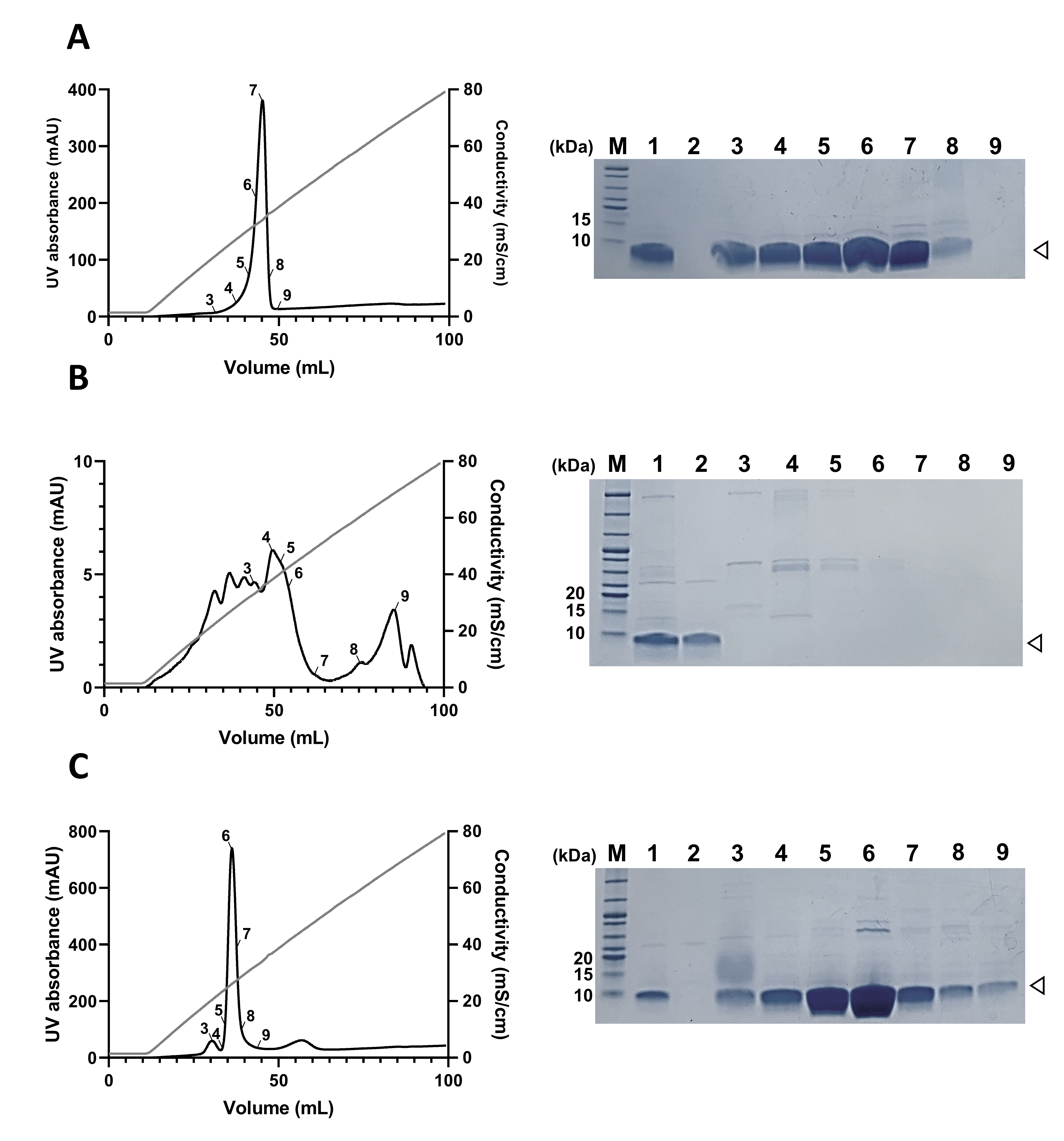

Supplement: Supplementary file 1 [file jmb-35-e2411076-supple.tif]
